# Correction: Novel insights in endocrine and metabolic pathways in sepsis and gaps for future research

**DOI:** 10.1042/CS-2021-1003C_COR

**Published:** 2022-07-04

**Keywords:** Critical Illness, Fasting, Hypercortisolemia, Ketogenesis, Sepsis, Tight glucose control

The authors of the original article would like to correct Figure 1 of their paper. In the revised Figure 1 presented here, two technical errors have been resolved. The growth hormone line (orange) during recovery phase should be a straight line and does not increase briefly one week after discharge. Secondly, the TSH/T4 line (green) should briefly rise upon recovery and normalizes thereafter. Both errors were the result of erroneous use of the vectorial drawing software.

**Figure 1:**
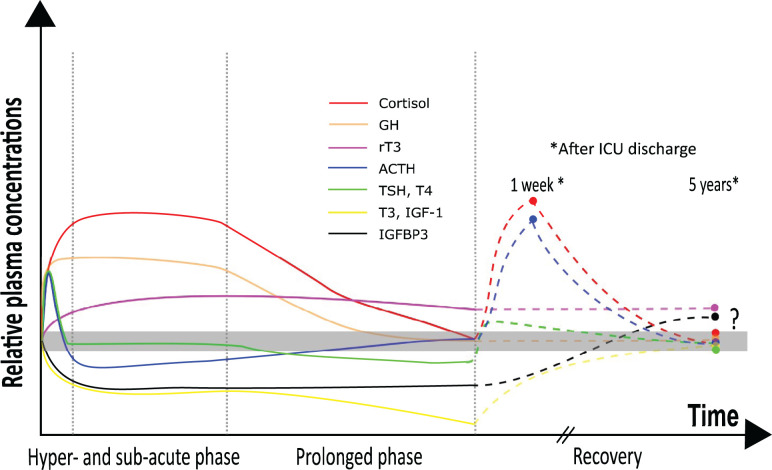
Neuroendocrine alterations during sepsis-induced critical illness and upon recovery Simplified cartoon depicting the current knowledge on the biphasic neuroendocrine responses to sepsis-induced critical illness and recovery. Solid color-coded lines show the trends in circulating hormone levels during the various phases of sepsis-induced critical illness (hyper-acute, sub-acute and prolonged phase). Solid dots show the observed alterations upon two time points in the recovery phase (two studies, investigating patients 1 week after ICU discharge for the HPA-axis hormones only and 5 years after ICU discharge for most neuroendocrine axes). Dotted color-coded lines show the potential trajectory in circulating hormone levels. Adapted with permission from “Teblick A, Langouche L, Van den Berghe G. Anterior pituitary function in critical illness. Endocr Connect. 2019;8:R131-R43.” [16].
